# How labor market institutions influence the relationship between exchange rate regimes and economic growth

**DOI:** 10.1371/journal.pone.0332492

**Published:** 2025-09-24

**Authors:** Vytautas Kuokštis, Muhammad Asali, Simonas Algirdas Spurga

**Affiliations:** 1 Vilnius University, Institute of International Relations and Political Science, Vilnius, Lithuania; 2 College of Management Academic Studies, Rishon LeZion, Israel; Southern Illinois University Carbondale, UNITED STATES OF AMERICA

## Abstract

How do exchange rate regimes affect economic growth? Although this topic has attracted considerable empirical attention, a definitive answer remains elusive. This study aims to advance our understanding by examining how labor market flexibility affects the link between exchange rate regimes and growth. Using a panel of 194 countries from 1970 to 2019, we find that in developing economies, fixed exchange rate regimes hinder growth when labor markets are highly rigid but boost growth when labor markets are highly flexible. We also demonstrate that this relationship varies depending on which labor market flexibility indicator is used, reflecting the differences in each measure’s geographic and temporal coverage.

## Introduction

How do exchange rate regimes affect macroeconomic performance, and in particular long-run economic growth? This question is highly relevant in international economics, development, and international political economy. Interestingly, there seems to be a divide between policymakers and economists regarding the impact of foreign exchange policies on economic growth. While policy discourse often holds that a lower exchange rate will encourage economic growth, economists tend to be skeptical that relative currency prices are a fundamental long-run growth driver. For many economists, the exchange rate is an endogenous variable, whose contribution to growth may be difficult to disentangle [1]. Addressing the impact of exchange rate regimes on long-run growth could have profound implications for both exchange rate policy design and the international monetary system. Yet despite a vast empirical literature on this nexus, no definitive consensus has emerged. Notably, labor market institutions remain underexplored within empirical research.

The optimum currency area (OCA) theory provides a foundational lens for assessing a country’s exchange rate choices and clarifying the role of labor market institutions, even though the theory was first designed to assess the benefits and costs of joining a currency union [2–4]. Based on OCA theory, one can predict that economies with more flexible labor markets will be better equipped to handle the constraints of a fixed exchange rate because labor market flexibility constitutes an alternative channel for adjustment. Flexible labor markets enable the real exchange rate (RER) to adjust when changes in the nominal rate are ruled out.

The standard model thus predicts that a fixed exchange rate is better for growth in countries with more flexible labor markets. Yet, despite these well-established theoretical expectations, to our knowledge, no large-N study has empirically tested them.

We address this gap by analyzing whether fixed exchange rate regimes hinder growth when labor market flexibility is low but foster it when flexibility is high. Kuokštis, Asali, and Spurga previously examined how labor market flexibility shapes exchange rate regime *choices* [4] and how it helps countries with fixed exchange rates recover from global recessions [5]. In this article, we focus on the dependent variable of long-run economic growth using static fixed-effects panel models and dynamic panel estimators based on the generalized method of moments (GMM).

Our results suggest that labor market flexibility plays a moderating role in the relationship between exchange rate regimes and growth, though with important caveats. The findings are statistically significant only for developing countries and depend on the flexibility measure and sample used. Using the CBR Labour Regulation Index [6], we find that fixed exchange rates hinder growth in rigid (more regulated) labor markets but increase it in economies with flexible (less regulated) labor markets. These effects are especially pronounced in the long run according to the dynamic panel analysis. In contrast, results using the Fraser Institute’s Labor Market Regulation indicator [7] are not significant, likely due to its more limited coverage. However, imputing the missing Fraser data yields results consistent with those based on the CBR Index.

The next section surveys the literature on how exchange rate regimes affect economic growth. Drawing on OCA theory, we then explain why labor market flexibility can shape this relationship. We next outline our data sources and methodological choices, present the empirical results, and conclude with their academic and policy implications.

## Previous literature

Which exchange rate regime is most conducive to sustained economic growth? The prevailing view in economics is that there is no one-size-fits-all prescription [8]. Both fixed and floating exchange rates entail distinct costs and benefits [4,9]. Fixed exchange rate regimes can provide important advantages by offering greater predictability and stability, thereby fostering international economic integration. Because foreign trade and investment tend to enhance efficiency and support long-run growth, such stability can translate into higher growth under a rigid currency regime. In macroeconomically unstable settings, fixed rates can also help build credibility, anchor expectations, and reduce inflationary pressures. Maintaining a credible peg requires macroeconomic policies consistent with the fixed rate, which constrains inflationary tendencies. By binding themselves to these constraints, policymakers signal a commitment to stability, boosting confidence in the economy, lowering inflation expectations, and improving the investment climate, all of which can promote growth.

Fixed exchange rate regimes, though praised for stability and policy discipline, can also impose substantial costs [4]. Chief among them is the loss of the nominal exchange rate as an adjustment tool: when economies face RER overvaluation or high unemployment, they cannot devalue to regain competitiveness. Under full capital mobility, the monetary policy trilemma [10] means that fixing the exchange rate also sacrifices independent monetary policy. Conventional levers—lowering interest rates in downturns or raising them to cool an overheating economy—become unavailable, curbing a country’s capacity to counter cyclical fluctuations [4]. In short, the fix restricts adjustment to domestic macroeconomic needs, which is the flip side of its stability benefits.

The empirical literature remains inconclusive about the effects of exchange rate regimes on economic growth, especially over the long run. Some studies, such as Edwards and Levy Yeyati [11], find that countries with more flexible regimes grow faster, because trade shocks are amplified under rigid ones. Others [12,13] argue that intermediate exchange rate regimes are most conducive to growth, as they preserve some monetary independence without sacrificing too much exchange rate flexibility. Meanwhile, Dubas, Lee, and Mark [14] report that the highest growth rates occur under *de facto* fixers.

Many studies distinguish the effects of exchange rate regimes by a country’s level of development. In developing economies, shallow financial markets, limited monetary credibility, and reliance on trade and capital inflows may favor exchange rate fixity. Evidence, however, is mixed. Husain, Mody, and Rogoff [15] find that flexibility boosts growth in advanced economies but yields no gains for developing ones, whereas Cruz-Rodríguez [16] reports higher growth in developing countries with fixed regimes. Bohl, Michaelis, and Siklos [17] conclude that fixed regimes work best for emerging markets, while crawling pegs deliver the greatest growth in the G20. By contrast, Bleaney and Francisco [18] show that hard pegs suppress growth relative to soft pegs and floats, and several studies [19–21] link greater flexibility in developing economies to faster growth and lower output volatility.

Finally, some authors report no robust relationship between exchange rate regimes and growth. Ghosh, Gulde, Ostry, and Wolf [22] find no systematic differences in growth rates or output volatility across regimes in a sample of 136 countries during 1960–1990. Terrones [23] likewise finds no stable long-run link, though he notes advantages of flexible rates during recoveries from global crises. Domac, Peters, and Yuzefovich [24] reach similar conclusions for transition economies. In Baycan [25], the average treatment effect of floating regimes on growth is statistically insignificant. Bailliu, Lafrance, and Perrault [26] add that the presence of a monetary-policy anchor, rather than the regime type itself, matters for growth.

Overall, if any consensus exists, it could be described as a “null finding”: in aggregate, exchange rate regimes appear to have little effect on economic growth. A broad survey by Harms and Kretschmann [27] reinforces this ambivalence, consistent with monetary neutrality: the view that nominal variables do not shape real outcomes in the long run.

Given these considerations, it is evident that conflicting theoretical and empirical considerations of exchange rate regimes and growth nexus persist. We aim to contribute to this body of literature by empirically analyzing the moderating role of labor market flexibility in this relationship.

## Theory

OCA theory remains a central tool for judging whether a country should adopt a fixed or flexible exchange rate regime. Depending on a given country’s conditions, one policy may be more efficient than the other. Key factors [28–31] include the symmetry of business cycles among countries that fix their currencies, the depth of economic and fiscal integration, the degree of capital and labor mobility, and labor market flexibility. The more symmetric the cycles, the deeper the integration, and the higher the mobility and flexibility of factors of production, the stronger the case for pegging the exchange rate. A country also benefits more from a fixed regime if it faces relatively few real shocks, such as terms-of-trade shocks. Within this framework, labor market flexibility is critical [4,31,32].

### Defining labor market flexibility

According to OCA theory, labor market flexibility is pivotal in determining whether a country gains or loses from adopting a fixed versus a flexible exchange rate. We define labor market flexibility broadly, as encompassing both wage flexibility and labor mobility [4]. It is the capacity of the labor market to reallocate labor and adjust wages efficiently when shocks occur. A more flexible market can therefore return to equilibrium more quickly after a disturbance [33].

Empirical research shows that labor market regulations support both wage flexibility and labor mobility, helping markets absorb shocks. Measures that proxy wage rigidity—such as employment protection legislation (EPL)—are linked to a muted wage response to unemployment changes [34]. Furthermore, research demonstrates that wage stickiness is substantially higher in formal than in informal labor markets [35].

Like wages, labor reallocation can depend on the flexibility of labor market institutions. Rigid regulations can limit firms’ ability to adjust their workforces, reducing both job creation and separations [36]. Haltiwanger, Scarpetta, and Schweiger [37] find that distortionary labor rules significantly slow job reallocation across a sample of developed and emerging economies.

Because labor market institutions are known to shape labor market outcomes, we use them to define labor market flexibility.

### Labor market flexibility, exchange rate regimes, and economic growth

To see why labor market flexibility matters, recall the key weakness of a peg. Fixing the nominal exchange rate eliminates both the exchange-rate tool and, under full capital mobility, independent monetary policy. If a fixing country faces loss of competitiveness or high unemployment, unless it is willing to abandon the peg, it must turn to “internal devaluation” [38,39]. Internal devaluation means the reduction of domestic real wages and salaries, which in turn can allow a lower price level and thus the depreciation of the RER. Internal devaluation can therefore substitute for nominal exchange rate (external) devaluation. The RER depreciates via lower domestic prices instead of a lower nominal exchange rate, since the RER is a function of the domestic price level, the foreign price level, and the nominal exchange rate.

Although internal devaluation can theoretically match the adjustment achieved by external devaluation, it is generally considered slower and less efficient because of nominal rigidities and price stickiness [4]. Whereas the exchange rate can depreciate (or be devalued) overnight, wages and prices may take much longer to fall to comparable levels of competitiveness. As a result, the process is often politically fraught and painful.

Nevertheless, the capacity to implement internal devaluation is not necessarily the same across countries. Specifically, it can depend on the degree of labor market flexibility. When wages adjust quickly to productivity changes, firms can cut prices sooner and remain competitive internationally. Evidence shows that higher hiring costs deepen output losses in recessions [40] and that flexible labor markets accelerate economic recoveries [41]. Wage declines also depreciate the RER, improving competitiveness and helping the current account move toward equilibrium [42]. Rigid labor market institutions may slow this process, and studies link greater flexibility to lower RER levels [43].

Furthermore, labor mobility enhances its reallocation towards more productive sectors and companies [44]. In open economies, higher productivity growth allows nominal wages to rise without increasing unit labor costs, thereby preventing upward pressure on prices. This supports a more competitive RER without requiring nominal depreciation, strengthening export performance and improving the external balance [45,46].

Overall, greater labor market flexibility should let a fixing country adjust faster and with less pain, reducing the cost of giving up monetary and nominal exchange rate policy. Such economies can thus keep the certainty and integration benefits of a fixed exchange rate while bearing lower adjustment costs, making the net effect on growth more likely to be positive.

By contrast, countries with rigid labor markets lack alternative adjustment mechanisms and are therefore likely to suffer under fixed exchange rate regimes. This logic underlies the International Monetary Fund’s (IMF) and European Central Bank’s (ECB) advice to countries that maintain pegs or belong to currency areas such as the euro area [47,48]. It can also explain why countries with higher labor market flexibility are more likely to *choose* more rigid exchange rate regimes [4].

The nexus between exchange rate regimes and labor market institutions in relation to macroeconomic performance remains insufficiently explored in the scholarly literature. Several studies have focused on the euro area, emphasizing how the institutional design of the Economic and Monetary Union (EMU)—a form of a hard peg—interacts with persistent national differences in labor market structures. By stripping away nominal flexibility while leaving divergent wage-setting systems intact, the euro fostered sizable North–South imbalances that built up before the Global Financial Crisis [49–51]. Extending this line of thought beyond euro area, Kuokštis and Spurga [9] show that exchange rate rigidity produces trade and current-account surpluses when labor markets are flexible but deficits when they are rigid, implying that only fixers with flexible labor markets gain competitiveness. Yet no large-N study has tested how this interaction shapes long-run economic growth; only one study examines how labor market flexibility aids post-crisis recoveries under fixed rates [5].

We therefore put forward the following hypothesis:

**H1** *The effe*ct of fixed exchange rate regimes on economic growth will be negative at low levels of labor market flexibility and positive at high levels of labor market flexibility.**

## Data

We compile an annual panel dataset covering 194 countries, for the years 1970–2019, comprising of exchange rate regimes, demographic data, labor market regulations, and global economic variables, although the actual working samples are smaller due to missing values for some variables from the different sources of the assembled data *De facto* exchange rate regime data is sourced from Ilzetzki, Reinhart, and Rogoff [52]. The flexibility of the exchange rate regime is defined on a fine scale (1–13, 1 being a currency union or no separate legal tender, and 13 denoting a freely floating regime), a coarse scale (1–4), and a dichotomous scale: fixed and floating (0–1). Growth rates were calculated from World Bank Group real GDP per capita data (World Development Indicators).

### Measuring labor market flexibility

Our primary measure of labor market flexibility is based on the Center for Business Research (CBR) Labour Regulation Index data introduced by Adams et al. [6]. Our assumption is that the degree of employment regulations protecting workers—the key concept captured by CBR Labour Regulation Index—serves as a good proxy for labor market rigidity (or inversely, flexibility). For a robustness test, we use the Fraser Institute’s Labor Market Regulation indicator [7]. Both Indices have been previously used in literature to measure the influence of labor market regulations on economic outcomes: see [5,53–56] for CBR, and [4,9,40,57,58] for Fraser.

While the CBR Index relies on leximetric coding to track cross-national and intertemporal variation in legal rules, the Fraser indicator draws on a broader range of sources, including surveys. We employ both Indices to better address labor market flexibility’s multidimensional nature, encompassing both *de jure* regulation and *de facto* institutional diversity.

The CBR provides data on labor laws in 117 countries, for the period of 1970–2019, captured by 40 different variables reflecting a specific dimension of labor market regulation. It codes 40 legal indicators, each normalised on a 0–1 scale, where 0 denotes no or the lowest level of statutory protection and 1 denotes the highest. The indicators are averaged into five sub-indices: (A) regulation of different forms of employment; (B) working-time regulation; (C) dismissal protection; (D) employee representation; and (E) industrial action. Some indicators originate from cardinal legal quantities (e.g., maximum hours) while most begin as ordinal categories; after normalisation all appear as bounded numerical variables between 0 and 1.

To capture labor market flexibility, we invert the original CBR protection scores so that higher values correspond to lower regulatory intensity. Next, we aggregate these transformed scores within each regulatory category by summing the relevant indicators, generating five intermediate flexibility scores: $F_{A}=\sum_{i=\text{1}}^{\text{8}}F_{i},\;F_{B}=\sum_{i=\text{9}}^{\text{1}\text{5}}F_{i},\;F_{C}=\sum_{i=\text{1}\text{6}}^{\text{2}\text{4}}F_{i},\;F_{D}=\sum_{i=\text{2}\text{5}}^{\text{3}\text{1}}F_{i},\;F_{E}=\sum_{i=\text{3}\text{2}}^{\text{4}\text{0}}F_{i}$. We then use principal component analysis (PCA) on these five sub-indices to construct a composite measure of overall labor market flexibility. The resulting Index is rescaled to range from 0 to 10, where 10 denotes the most flexible (least regulated) labor market.

The composite Index is subsequently used in conjunction with exchange rate regime data to assess the effect of their interaction on economic growth. For robustness checks, we also show results based on alternative CBR-based definitions of labor market flexibility, namely CBR1, which is the predicted level from PCA on all forty individual subcomponents; and CBR2, which is the simple average of all (non-missing) forty subcomponents. We refer to our chosen composite Index explained earlier as CBR3.

The estimated correlation coefficients (and p-values) between the different definitions of CBR measures are as follows: $r_{cbr\text{1},cbr\text{2}}=\text{0}.\text{9}\text{5}\text{6}\;(<\text{0}.\text{0}\text{0}\text{1}),\;r_{cbr\text{1},cbr\text{3}}=\text{0}.\text{9}\text{6}\text{2}\;(<\text{0}.\text{0}\text{0}\text{1}),\;r_{cbr\text{2},cbr\text{3}}=\text{0}.\text{9}\text{9}\text{7}\;(<\text{0}.\text{0}\text{0}\text{1})$.

**Fig 1:**
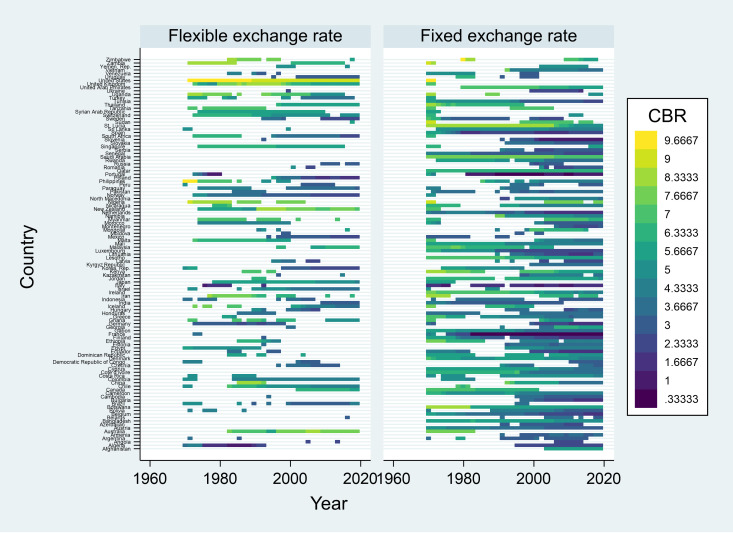
Labor market flexibility (CBR) by country, year, and exchange rate regime. Notes: Data for de facto exchange rate regimes from Ilzetzki, Reinhart, and Rogoff [52]. Data for labor market flexibility from Adams et al. [6].

Fig 1 shows the level of labor market flexibility, measured with the CBR Index defined as above (CBR3), across countries and over years of the working data.

Fig 1 illustrates that both exchange rate regimes and labor market flexibility exhibit a temporal variation across a number of countries. Overall, it suggests a general trend of decreasing labor market flexibility, with some countries imposing more regulations over time.

Table 1 shows summary statistics of the main variables for some years spanning the study period (1971, 2000, and 2019), as well as for the entire sample, by the exchange rate regime in the countries. Both the global economic growth rate and labor market flexibility have fallen, especially over the past two decades. Shifts in the demographic and economic profiles of countries with fixed versus floating rate regimes suggest that many have switched regimes during this period.

**Table 1:** Summary statistics of the main variables, by exchange rate regime (1971, 2000, 2019, and the entire sample).

		Flexible exchange rate				Fixed exchange rate			
		Mean	SD	Min	Max	Mean	SD	Min	Max
**1971**	Growth (%)	2.58	6.03	−13.31	8.64	3.99	5.01	−6.63	23.34
	Labor market flexibility	5.3	1.58	3.96	9.71	6.04	1.66	1.59	9.79
	GDP per capita (1000s)	2.82	2.53	0.78	9.63	8.93	9.78	0.14	36.11
	Population (millions)	28.49	25.69	4.69	98.77	34.39	83.65	0.3	570
	Population growth (%)	2.2	0.36	1.58	2.75	2.01	1.2	−0.42	4.65
**2000**	Growth (%)	3.84	3.66	−4.18	18.91	3.24	3.05	−4.36	10.19
	Labor market flexibility	5.01	1.61	2.92	9.3	4.26	1.7	0.04	8.12
	GDP per capita (1000s)	16.74	19.97	0.51	73.75	13.78	18.55	0.26	92.53
	Population (millions)	51.3	67.09	0.39	282.16	58.09	202.82	0.16	1262.65
	Population growth (%)	1.03	1.14	−1.94	3.14	1.31	1.2	−1.18	5.58
**2019**	Growth (%)	1.06	1.6	−1.72	4.48	2.2	2.44	−4.23	8.21
	Labor market flexibility	4.28	1.88	1.75	9.3	3.68	1.45	0.52	7.81
	GDP per capita (1000s)	30.02	25.31	1.94	87.12	16.2	20.75	0.49	107.35
	Population (millions)	114.01	275.2	0.36	1383.11	52.25	169.35	0.18	1407.74
	Population growth (%)	0.99	0.7	−0.14	2.2	1.12	1.11	−1.15	3.93
**Sample**	Growth (%)	2.21	4.02	−28.44	24.52	2.7	4.51	−29.92	36.56
	Labor market flexibility	5.09	1.93	0.83	10	4.38	1.76	0	9.79
	GDP per capita (1000s)	17.96	19.95	0.14	87.12	13.05	17.49	0.14	113.97
	Population (millions)	62.47	150.12	0.28	1383.11	49.39	164.75	0.12	1407.74
	Population growth (%)	1.5	1.12	−1.94	6.34	1.57	1.62	−2.26	19.36

*Notes*: GDP per capita is in thousands of constant 2015 US dollars.

## Methodology

We study the effect of the exchange rate regime on economic growth, and how it is moderated by the flexibility of the labor market. The benchmark relationship therefore is:


$$g_{it}=\alpha_{i}+\beta_{\text{1}}FER_{it-\text{1}}+\beta_{\text{2}}LMF_{i,t-\text{1}}+\beta_{\text{3}}FER_{it-\text{1}}\times LMF_{i,t-\text{1}}+\gamma \text{ln}\left(GDPPC_{i,t-\text{1}}\right)+X_{it}\delta +\phi t+\varepsilon_{it}$$ (1)

In the equation, $g_{it}$ represents the growth rate of the real per-capita GDP of country *i* in year *t*. We use the exact definition of the growth rate ($g_{t}=\Delta GDPPC/GDPPC_{t-\text{1}}$) rather than the approximation usually used (which is ΔlnGDPPC). This choice, however, has virtually no effect on the results of this study. Fixed effects estimation allows capturing the country individual fixed effects $\alpha_{i}$. The variable *FER* stands for the fixity of the exchange rate regime. Our main analysis and results pertain to using the dichotomous measure of exchange rate regimes: FER=0 if the exchange rate regime is flexible, and FER=1 if it is fixed. In our robustness checks, we use the fine scale [from 1 to 13] of exchange rate regimes as provided by Ilzetzki, Reinhart, and Rogoff [52].

For the purpose of this study, we reverse the definition of the exchange rate regime categories, so that level 1 stands for freely floating exchange rate, and level 13 for very hard fix (currency union or no separate legal tender). It is important to emphasize that, except for population size and population growth, the independent variables of interest are included in the analysis in their lagged (t-1) form. Population size serves as a contemporaneous control variable for country size. This is standard in literature as the effects of explanatory variables on the outcome variable typically take time to materialize and are not observed immediately. We therefore assume that a change in policy in year (t-1) might affect GDP by the following year, which is captured by growth in year (t).

In a panel data setting with both cross-sectional units (countries) and time periods (years), pooled OLS model can produce biased results due to unobserved heterogeneity. Even when OLS is unbiased, it is typically inefficient, making fixed-effects or random-effects estimation appropriate depending on the correlation structure. To assess this, we conducted a Hausman specification test, which strongly rejected the random-effects model. The test yielded a Chi-squared statistic of 84.91 with a p-value approaching zero, providing robust evidence against the null hypothesis that the random-effects estimator is both consistent and more efficient than the fixed-effects alternative. Therefore, we chose the fixed-effects model.

*LMF* is the used measure of the labor market flexibility, as defined in the Data section of this study. The vector X includes time-variant control variables like the population size and population growth (in earlier working versions of this study, we included additional regressors like polity and the Chinn-Ito Index of capital account openness; their inclusion had virtually no effect on the study results.) Index φt captures a linear time trend, and $\varepsilon_{it}$ is the error term, a mean-zero growth innovation.

The total effect of the exchange rate regime on the growth rate of the country is given by:


$$\frac{\partial g_{t}}{\partial FER_{t-\text{1}}}=\beta_{\text{1}}+\beta_{\text{3}}LMF_{t-\text{1}},$$

hence, it depends on the level of labor market flexibility. Of particular interest is the coefficient of the interaction term $\beta_{\text{3}}$: A positive coefficient means that more flexible labor markets render the effect of fixed exchange rate regimes on economic growth more positive (or less negative).

We investigate this relationship with different specifications, and for different groups of countries (developing versus developed). We then augment the model to a dynamic panel, that allows us to study the long-run effects. In particular, we estimate the following dynamic panel model:


$$g_{it}=\alpha_{i}+\pi g_{it-\text{1}}+\beta_{\text{1}}FER_{it-\text{1}}+\beta_{\text{2}}LMF_{i,t-\text{1}}+\beta_{\text{3}}FER_{it-\text{1}}\times LMF_{i,t-\text{1}}+\gamma \text{ln}\left(GDPPC_{i,t-\text{1}}\right)+X_{it}\delta +\phi t+\varepsilon_{it}$$ (2)

We first estimate this model by fixed-effects regression, as a benchmark, and then use a GMM estimation method, proposed by Arellano and Bond [59], to address the potential inconsistency in the estimates due to the inclusion of the lagged dependent variable that is correlated with the unobserved individual (panel-level) effects. In this dynamic framework, while the short-run effect of the exchange rate regime is captured by $\beta_{\text{1}}+\beta_{\text{3}}LMF$, as before, the long-run effect is captured by:


$$\text{long}-\text{run effect of Fixed ER on growth}=\frac{\beta_{\text{1}}+\beta_{\text{3}}LMF}{\text{1}-\pi }$$

This captures the steady-state effect of exchange rate fixity on economic growth at different levels of labor market flexibility. Importantly, this measure does not imply a fixed level of economic growth in the steady state—rather, it measures the *effect* of exchange rate fixity on any growth level in the long run.

## Results

We first analyze the effect of fixed exchange rate regimes on economic growth (measured as real per-capita GDP growth), as moderated by labor market flexibility, using static panel regression analysis. We then investigate these effects in the long run using a dynamic panel regression framework.

### Growth regressions: static panel analysis

Table 2 reports the estimation results for the effect of fixed exchange rate regimes on growth in the static model, using three variants of the CBR measure of labor market flexibility. CBR1 represents the CBR Index constructed from PCA of the forty individual subcomponents of the Index; CBR2 represents the simple mean of the forty subcomponents; and CBR3, the benchmark specification, is constructed from PCA of the five dimensions of the Index’s subcomponents.

**Table 2:** The effect of exchange rate regimes on economic growth taking into account labor market flexibility.

	(1)	(2)	(3)	(4)	(5)
	Full	Restricted	CBR1	CBR2	CBR3
Fixed exchange rate ($FER_{t-\text{1}}$)	0.829***	0.547**	−1.321**	−1.683**	−1.606**
	(0.260)	(0.276)	(0.606)	(0.746)	(0.699)
Labor market flexibility ($LMF_{t-\text{1}})$			−0.054	−0.118	−0.125
			(0.158)	(0.175)	(0.169)
$FER_{t-\text{1}}\times LMF_{t-\text{1}}$			0.393***	0.462***	0.457***
			(0.133)	(0.156)	(0.151)
Log population	−1.582*	−1.681	−2.023**	−1.942*	−1.910*
	(0.843)	(1.016)	(1.000)	(0.994)	(0.988)
Population growth	−0.483***	−0.729***	−0.734***	−0.734***	−0.735***
	(0.135)	(0.144)	(0.146)	(0.145)	(0.146)
Log GDP per capita (lag)	−3.081***	−3.065***	−3.186***	−3.165***	−3.160***
	(0.588)	(0.625)	(0.586)	(0.591)	(0.591)
Time trend	Yes	Yes	Yes	Yes	Yes
Observations	7962	4066	4066	4066	4066
$R^{\text{2}}$ within	0.036	0.054	0.059	0.060	0.060

*Notes:* The dependent variable is the annual growth rate of real GDP per capita. CBR1 refers to the CBR Index of labor market flexibility (normalized to a 0–10 scale, with 10 denoting the most flexible labor market) constructed using PCA of the 40 subcomponents of the CBR Index; CBR2 is based on the simple average of these 40 subcomponents, while CBR3 is derived from PCA of the five main sub-indices of the CBR Index. Robust standard errors, clustered at the country level, are reported in parentheses.

*** p < .01, ** p < .05, * p < .1

Column 1 of Table 2 reports the estimation results for the simplest unconstrained sample with an unconditional specification, where we use fixed-effects regression to estimate the effect of lagged fixed exchange rate regimes on economic growth, without taking into account labor market flexibility. The results indicate a positive coefficient of 0.83 and statistically significant effect of fixed exchange rate regimes on economic growth. The magnitude of this effect diminishes when we restrict the sample to observations with non-missing values for labor market flexibility (Column 2).

When incorporating labor market flexibility, as measured by CBR1, CBR2, and CBR3 in Columns 3, 4, and 5, respectively, we find a large and highly statistically significant interaction effect between labor market flexibility and exchange rate fixity on economic growth. The estimation results across all variations of the CBR Index hover around a partial effect of 0.39–0.46.

The main finding of this study, as evidenced by Table 2, is that the effect of fixed exchange rate on economic growth is conditional on labor market flexibility. Specifically, an increase in labor market flexibility amplifies the effect of exchange rate fixity on economic growth by a statistically significant 0.457 percentage points (Column 5). Notably, even the direction of the effect of exchange rate fixity depends on labor market flexibility: the effect is −1.606 percentage points in economies with very rigid labor markets (LMF = 0), compared to a substantial positive effect of 2.97 (=−1.606+0.457×10) percentage points in economies with highly flexible labor markets (LMF = 10). The corresponding total effects for highly flexible labor markets under CBR1 and CBR2 specifications are 2.61 and 2.94, respectively.

Table 3 reports the total effect of fixed exchange rates on growth, based on the Table 2 results, estimated at three labor market flexibility levels: 0 (rigid), 5 (medium), and 10 (highly flexible). Without accounting for labor market flexibility, fixed exchange rates are expected to have a positive effect on economic growth, as shown in Column 1 of Table 3. Columns 2–4, which employ different variants of the CBR Index, demonstrate that the effect of fixed exchange rate regimes on economic growth is conditional on the degree of labor market flexibility: fixed exchange rates have a negative effect on economic growth in economies with very rigid labor markets, and a large, positive effect on growth in economies with highly flexible labor markets.

**Table 3:** Total effect of fixed exchange rate regimes on economic growth at different levels of labor market flexibility.

	Restricted	CBR1	CBR2	CBR3
	0.547** (0.276)			
At Labor market flexibility = 0		−1.321** (0.606)	−1.683** (0.746)	−1.606** (0.699)
At Labor market flexibility = 5		0.646** (0.262)	0.627** (0.256)	0.681*** (0.262)
At Labor market flexibility = 10		2.614*** (0.812)	2.937*** (0.893)	2.968*** (0.891)

*Notes:* Column heads refer to the respective specifications in Table 2. Robust standard errors, clustered at the country level and calculated using the delta method, are reported in parentheses.

*** p < .01, ** p < .05, * p < .1

**Fig 2:**
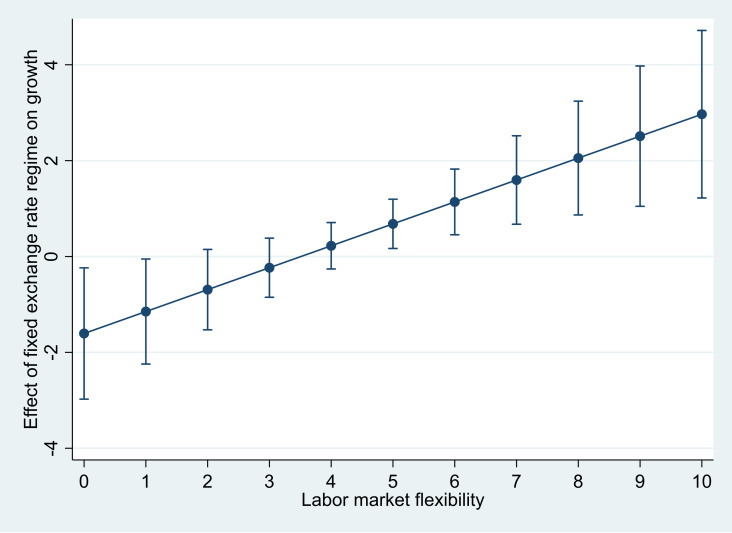
Average marginal effects of fixed exchange rate regimes on growth (with 95% CIs). Notes: Based on Column 5 of Table 2.

Fig 2 shows these findings across all labor market flexibility levels, from 0 to 10, using the CBR3 Index, with 95% confidence intervals. The upward-sloping line demonstrates that the effect of fixed exchange rate regimes on economic growth increases with labor market flexibility.

### Long-run effects of fixed exchange rate regimes: dynamic panel analysis

Table 4 reports the results analogous to those of the static model but employs a dynamic panel setting. This is achieved by incorporating the lagged dependent variable.

**Table 4:** Dynamic panel analysis of fixed exchange rates and economic growth taking into account labor market flexibility.

	(1)	(2)	(3)
	Unconditional	FE	IV-FE
Fixed exchange rate ($FER_{t-\text{1}}$)	0.292	−1.291**	−2.853**
	(0.226)	(0.549)	(1.301)
Labor market flexibility ($LMF_{t-\text{1}})$		−0.107	−1.031***
		(0.131)	(0.357)
$FER_{t-\text{1}}\times LMF_{t-\text{1}}$		0.337***	0.729***
		(0.114)	(0.255)
Lagged growth rate	0.273***	0.269***	0.139***
	(0.032)	(0.032)	(0.030)
Log population	−1.744**	−1.911**	−6.484***
	(0.769)	(0.751)	(1.998)
Population growth	−0.699***	−0.704***	−0.933***
	(0.127)	(0.128)	(0.159)
Log GDP per capita (lag)	−3.165***	−3.240***	−10.290***
	(0.504)	(0.484)	(1.802)
Time trend	Yes	Yes	Yes
Observations	4045	4045	3868
R-squared	0.137	0.140	0.050

*Notes:* The dependent variable is the annual growth rate of real GDP per capita. Labor market flexibility is measured by the CBR Index (CBR3). Four lags of the dependent variable are used as instruments in the Arellano-Bond procedure for estimating instrumental variables fixed-effects models (IV-FE). Robust standard errors, clustered at the country level, are reported in parentheses.

*** p < .01, ** p < .05, * p < .1

Column 1 reports the effect of fixed exchange rate regimes on growth without accounting for labor market flexibility. This effect is positive but statistically insignificant, although the endogeneity of the lagged dependent variable renders these estimates inconsistent. Column 2 accounts for labor market flexibility, and the results indicate a positive interaction effect between labor market flexibility and fixed exchange rate regimes on economic growth. Given the endogeneity of the lagged dependent variable, we employ a more consistent GMM estimator following Arellano and Bond [59] in Column 3. The estimation results from the dynamic panel models in Table 4 confirm our earlier findings, as the effect of exchange rate fixity on growth depends on labor market flexibility. This effect is positive, statistically significant, and persistent. In the long-run, exchange rate fixity promotes growth in economies with flexible labor markets but hinders growth in economies with highly regulated labor markets.

Table 5 reports total short-run and the total long-run effects of exchange rate fixity on growth across different labor market flexibility levels, based on the Table 4 estimation results.

**Table 5:** Dynamic panel analysis of the total effect of fixed exchange rate regime on economic growth at different labor market flexibility levels: short-run and long-run effects.

	Unconditional		FE		IV-FE	
	SR	LR	SR	LR	SR	LR
	0.292 (0.226)	0.401 (0.310)				
At Labor market flexibility = 0			−1.291** (0.549)	−1.766** (0.758)	−2.853** (1.301)	−3.312** (1.524)
At Labor market flexibility = 5			0.395* (0.219)	0.542* (0.297)	0.790* (0.491)	0.918* (0.565)
At Labor market flexibility = 10			2.081*** (0.670)	2.847*** (0.917)	4.433*** (1.426)	5.147*** (1.656)

*Notes:* Column heads refer to the respective specifications in Table 4. Robust standard errors, clustered at the country level and calculated using the delta method, are reported in parentheses.

*** p < .01, ** p < .05, * p < .1

At the lowest labor market flexibility levels, the effects of exchange rate fixity on growth are negative both in the short-run and in the long-run. At the highest labor market flexibility levels, the effect of fixed exchange rate on growth is positive and highly statistically significant; this effect is larger in the long run than in the short run.

### Developing and developed countries

We split our sample by development status (developing versus developed countries) and re-examine our findings using both the static and the dynamic model specifications. The results are summarized in Table 6.

**Table 6:** The effect of fixed exchange rate regimes on economic growth in developing and developed countries.

	Developing		Developed	
	Static	Dynamic	Static	Dynamic
Fixed exchange rate ($FER_{t-\text{1}}$)	−2.314**	−3.982***	0.504	0.472
	(1.064)	(1.529)	(0.564)	(0.622)
Labor market flexibility ($LMF_{t-\text{1}})$	−0.367	−1.399***	0.298***	0.139
	(0.266)	(0.461)	(0.109)	(0.107)
$FER_{t-\text{1}}\times LMF_{t-\text{1}}$	0.622***	0.968***	−0.090	−0.068
	(0.207)	(0.313)	(0.119)	(0.114)
Log population	−5.218***	−12.373***	0.437	1.799*
	(1.752)	(2.794)	(1.430)	(1.050)
Population growth	−0.729***	−0.811***	−0.383*	−0.525***
	(0.168)	(0.143)	(0.201)	(0.172)
Log GDP per capita (lag)	−3.844***	−11.373***	−3.103***	−3.576***
	(0.724)	(1.753)	(0.777)	(0.749)
Time trend	Yes	Yes	Yes	Yes
Lagged growth rate		0.139***		0.321***
		(0.036)		(0.029)
Observations	2628	2478	1438	1390
R-squared	0.063	0.075	0.113	0.204

*Notes:* The dependent variable is the growth rate of real GDP per capita. Dynamic models are IV-FE models. Robust standard errors, clustered at the country level, are reported in parentheses.

*** p < .01, ** p < .05, * p < .1

Fixed exchange rate regimes have a highly statistically and economically significant effect on economic growth in developing countries only. This effect is negative under very rigid labor markets and positive under highly flexible labor markets. For developed countries, neither the short-run nor the long-run effects are significant.

The long-run effects, whether negative or positive, are more pronounced than the short-run effects. In developing economies with rigid labor markets, the short-run effect of fixed exchange rate regimes on economic growth is −2.314 (−3.982 from the dynamic estimation), compared to −4.624 in the long-run. At the highest labor market flexibility levels (LMF = 10), however, the effect in the short-run is a highly statistically significant +3.906 (SE 1.100), compared to +6.617 in the long-run.

## Robustness checks

We now examine the robustness of the results to different measures of exchange rate fixity, conducting this analysis using both static and dynamic models. We also compare the findings with results using a different measure of labor market flexibility: the Fraser Institute’s Labor Market Regulation indicator.

### Continuous measure of exchange rate fixity

First, we analyze the effects of exchange rate regimes on growth using a continuous scale rather than a dichotomous fixed-float measure. Specifically, we employ the variable “fixity of exchange rate,” which takes on values from 1 (free floating exchange rate) to 13 (no separate legal tender or currency union), replacing the dichotomous variable employed previously. Table 7 reports the results for both the static and the dynamic model specifications.

**Table 7:** Effect of exchange rate fixity on growth with a continuous exchange rate regime measure.

	Static	Static	Dynamic FE	Dynamic IV-FE
Fixed exchange rate ($FER_{t-\text{1}}$)	0.035	−0.265***	−0.208***	−0.544**
	(0.034)	(0.085)	(0.066)	(0.216)
Labor market flexibility ($LMF_{t-\text{1}})$		−0.321	−0.249	−1.341***
		(0.202)	(0.156)	(0.458)
$FER_{t-\text{1}}\times LMF_{t-\text{1}}$		0.065***	0.048***	0.116***
		(0.018)	(0.013)	(0.041)
Log population	−1.510	−1.999**	−2.046***	−6.983***
	(1.020)	(1.000)	(0.774)	(2.144)
Population growth	−0.735***	−0.728***	−0.696***	−0.929***
	(0.146)	(0.146)	(0.127)	(0.156)
Log GDP per capita (lag)	−3.045***	−3.217***	−3.295***	−10.501***
	(0.625)	(0.591)	(0.490)	(1.804)
Time trend	Yes	Yes	Yes	Yes
Lagged growth rate			0.268***	0.138***
			(0.032)	(0.029)
Observations	4066	4066	4045	3868
R-squared	0.053	0.061	0.141	0.052

*Notes:* Fixity of the exchange rate regime is a continuous scale of exchange rate regimes that lies between 1 (free floating) to 13 (currency union or no separate legal tender). All other variables, specifications, and estimation methods are the same as in Table 2 and Table 4. Robust standard errors, clustered at the country level, are reported in parentheses.

*** p < .01, ** p < .05, * p < .1

Table 7 demonstrates that the effect of exchange rate fixity on growth, and its interaction with labor market flexibility, does not depend on a dichotomous definition of exchange rate regimes. When measured on a continuous scale, exchange rate fixity exhibits a similar effect on economic growth, contingent upon labor market flexibility levels. The negative effect of exchange rate fixity on economic growth observed in economies with rigid labor markets becomes positive under more flexible labor markets. Moreover, the long-run effects are larger in magnitude than the short-run effects.

### Using an alternative measure of labor market flexibility

To test our hypothesis we also employ an alternative measure: the Fraser Institute’s Labor Market Regulation indicator [7]. This indicator is based on seven subcomponents: (i) hiring regulations and minimum wage, (ii) hiring and firing regulations, (iii) centralized collective bargaining, (iv) hours regulations, (v) mandated cost of worker dismissal, (vi) conscription, and (vii) regulations on the employment of foreign labor. The Fraser-based Labor Market Flexibility Index is constructed as the arithmetic average of all seven subcomponents, normalized to a 0–10 scale, where 10 represents the most flexible labor market (as in Kuokštis, Asali, and Spurga [4]).

**Fig 3:**
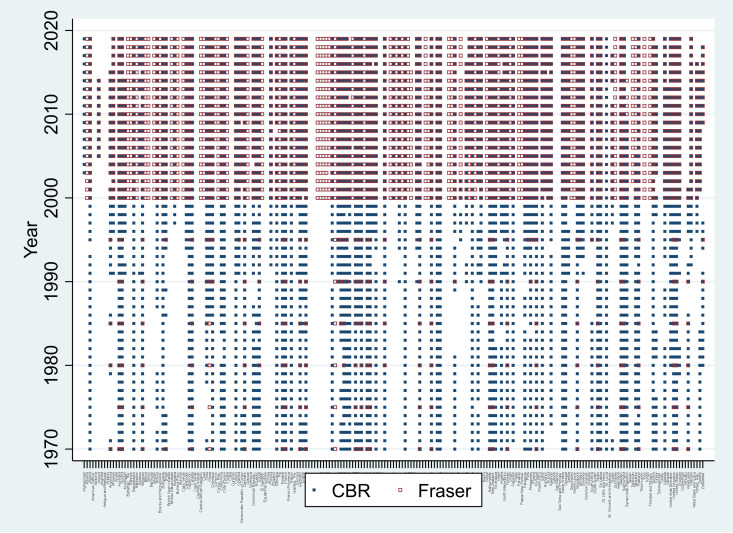
CBR and Fraser Index data availability. Notes: Data for CBR from Adams et al.[6]; data for Fraser from Gwartney, Lawson, Hall, and Murphy [7].

The Fraser-based indicator, however, has more limited temporal coverage, as data are unavailable for many years between 1970 and 2000; during this period, Fraser data are available only once every five years. For some countries, Fraser data are first available beginning in 2000. Fig 3 illustrates the temporal and cross-country coverage of the Fraser indicator relative to the CBR Index: outlined squares represent Fraser indicator availability, and filled squares represent CBR Index availability.

Despite the limited availability of the Fraser indicator for many country-year observations, we examine the robustness of our findings using this independent measure of labor market flexibility. We impute missing values for the Fraser indicator by extrapolating predicted values from a linear regression of available Fraser observations against the five overarching categories of the CBR Index ($F_{A}$ laws on employment, $F_{B}$ laws on working time, $F_{C}$ laws on dismissal, $F_{D}$ laws on employee representation, and $F_{E}$ laws on industrial action):


$$Fraser_{it}=\alpha +\beta_{a}F_{A}+\beta_{b}F_{B}+\beta_{c}F_{C}+\beta_{d}F_{D}+\beta_{e}F_{E}+\nu_{it}$$

Although the Fraser and CBR indices capture labor market flexibility through distinct methodologies, they exhibit a positive correlation. For the restricted sample (where both CBR and Fraser values are available), the correlation coefficient between the Fraser indicator and the CBR Index is 0.206 (positive and statistically significant, p-value <0.001). For the full CBR sample, where missing Fraser values were imputed as described above, the correlation coefficient between CBR and the Fraser is higher and highly statistically significant (0.355, p-value <0.001).

Table 8 presents the estimation results using both the CBR and Fraser indices. We report results for two samples: the “restricted sample,” which includes only observations with non-missing values for both indices; and the “full sample,” which includes all observations with a defined CBR Index, supplementing missing Fraser values with imputed data.

**Table 8:** The effect of fixed exchange rate regime on economic growth taking into account labor market flexibility with CBR and Fraser measures.

	Restricted sample		Full sample	
	Fraser	CBR	Fraser	CBR
Fixed exchange rate ($FER_{t-\text{1}}$)	0.811	−0.091	−1.867*	−1.606**
	(0.927)	(0.933)	(1.036)	(0.699)
Labor market flexibility ($LMF_{t-\text{1}})$	0.536***	−0.073	−0.162	−0.125
	(0.180)	(0.187)	(0.157)	(0.169)
$FER_{t-\text{1}}\times LMF_{t-\text{1}}$	-0.185	−0.055	0.391**	0.457***
	(0.145)	(0.165)	(0.176)	(0.151)
Log population	−0.667	−1.131	−1.581	−1.910*
	(1.385)	(1.376)	(1.020)	(0.988)
Population growth	−0.692***	−0.687***	−0.740***	−0.735***
	(0.212)	(0.213)	(0.147)	(0.146)
Log GDP per capita (lag)	−3.527***	−3.803***	−3.069***	−3.160***
	(0.928)	(0.952)	(0.621)	(0.591)
Time trend	Yes	Yes	Yes	Yes
Observations	2143	2143	4066	4066
$R^{\text{2}}$ within	0.098	0.092	0.056	0.060

*Notes:* The dependent variable is the annual growth rate of real GDP per capita. Fixed exchange rate is a dichotomous variable that takes the value 1 if the exchange rate is fixed, and zero otherwise. Labor market flexibility is a [0–10] scale Index for labor market regulation, with higher values corresponding to more flexible labor markets. Columns 1 and 2 were estimated for the “restricted sample,” where observations were available for both Fraser and CBR Indices. Columns 3 and 4 were estimated for all available data. However, since Fraser data have many missing values in earlier years, these have been imputed using extrapolated values from a linear regression of the Fraser indicator on the five building sub-indices of the CBR Index. Columns 1 and 3 use the Fraser (or imputed Fraser) Index as a measure of labor market flexibility. Columns 2 and 4 use the CBR (CBR3) Index as a measure of labor market flexibility. Regressions include fixed effects (FE) and a linear time trend. Robust standard errors, clustered at the country level, are reported in parentheses.

*** p < .01, ** p < .05, * p < .1

The first panel of Table 8 reports the estimation results from the restricted sample, and the second panel reports the estimation results from the full sample. Column 1 uses only the non-missing values of Fraser data. In this restricted sample the coefficient of the fixity-flexibility interaction term is slightly negative but not statistically significant. Using the CBR Index, as in Column 2, yields similar results, suggesting that labor market flexibility does not have a significant effect on the relationship between exchange rate fixity and economic growth.

Using the whole available sample on CBR, however, overturns this result. Column 4, which uses all available data on CBR, replicates our findings from Column 5 of Table 2, confirming that the effect of fixed exchange rates on growth is a function of the flexibility of the labor market. Using both actual and imputed Fraser values for the full sample, as in Column 3, provides corroborating evidence. Therefore, when using the “augmented” Fraser indicator as a measure of labor market flexibility, we find that the effect of exchange rate fixity on economic growth is an increasing function of labor market flexibility; the total effect is negative (−1.9) in rigid labor markets but positive and economically and statistically significant (2.0) in flexible labor markets.

## Conclusions

In this article, we analyze the moderating role that labor market flexibility plays in the relationship between currency regimes and economic growth. There are strong theoretical reasons to expect this moderating effect to be important. Based on OCA theory, we hypothesize that fixed exchange rates promote growth under flexible labor markets but hinder it under rigid ones.

Our empirical results suggest that such a moderating relationship is important, although there are significant caveats. First, the results are statistically significant for developing countries only. Second, the significance of the findings depends on the measure of labor market flexibility and the sample used. In this study, we focus on two alternative measures of labor market flexibility: the CBR Labour Regulation Index by Adams et al. [6] and, for robustness tests, the Fraser Institute’s Labor Market Regulation indicator [7].

We find a significant and economically substantial effect using the CBR Index. Fixed exchange rate regimes are found to have a negative effect on economic growth in economies with highly rigid labor markets, and a large positive effect in economies with highly flexible labor markets. Employing a dynamic panel analysis, we report long-run effects that are even more pronounced than the short-run effects.

However, we do not find significant results using the Fraser indicator for the subsample of countries-years for which Fraser data are available. The difference likely stems from the differing geographic and time coverage of the two indices, as the CBR Index encompasses a larger sample both temporally and geographically. Imputing values for the missing Fraser data using extrapolation from a linear conditional expectation model renders the results based on the Fraser data statistically and economically significant, similar to the main findings using the CBR Index.

More broadly, labor market flexibility is a contested concept theoretically and poses significant measurement challenges. Both measures of labor market flexibility employed in our study are legitimate, and both have been used in the previous literature. One takeaway is that researchers should be mindful of the different conclusions that can be reached using different operationalizations of labor market flexibility, which also applies to other important macroeconomic outcomes. Furthermore, our findings underscore that the labor market-exchange rate nexus may be context-dependent; we showed that the different conclusions reached using the CBR measure stem from its different sample coverage compared to the Fraser data. Moreover, the results hold for developing, but not developed, countries.

Our results have implications for policy. Exchange rate regime choice constitutes an important policy lever for developing and emerging economies reforming their monetary frameworks. Countries are often advised to transition gradually towards floating exchange rate regimes, as exemplified by the latest IMF program in Argentina [60]. We show that retaining a fixed exchange rate may not necessarily be disadvantageous compared to a floating regime, particularly when labor markets are sufficiently flexible. This introduces an important dimension to the policy debate on the macroeconomic benefits of exchange rate regime choice, highlighting the potential role of labor market institutions in evaluating the trade-offs involved.

Furthermore, our findings underscore the importance of institutional complementarities in macroeconomic policy design. When formulating policy prescriptions, policymakers and advisors should consider exchange rate arrangements and labor market reforms as interdependent levers in shaping a coherent growth strategy. The effects of a specific exchange rate regime on growth may be contingent upon a country’s labor market institutions. Enhancing labor market flexibility may allow countries to better leverage the benefits of exchange rate fixity.

Finally, future research should explore the specific *mechanisms* driving the effects of the labor market flexibility-exchange rate interaction. The effect is likely mediated by changes in the RER and external competitiveness. Furthermore, associated effects on export growth are expected, especially for price-sensitive goods and services.
